# Rheumatism

**Published:** 1890-11-29

**Authors:** 


### THE LONDON POST-GRADUATE COURSE AT THE PADDINGTON INFIRMARY.

### RHEUMATISM.

Dr. Cheadle, in commencing, said the infirmary was full of interesting cases of so many diseases that he found it somewhat difficult to choose a subject for his lecture, but after consideration he had determined to speak of the various forms of rheumatism and gout. He proceeded as follows: There are four classes of rheumatic affections, namely: (1) True rheumatism; (2) gonorrheal rheumatism; (3) gout; and (4) rheumatoid arthritis, osteo-arthritis, or rheumatic gout. True rheumatism was often styled rheumatic fever, but rheumatic fever really referred to a high expression of the disease, and especially when it occurred in adults. One great point about rheumatism was that the morbid changes produced by the rheumatic poison, whether this was chemical or the result of a micro-organism, were very widespread over the body; in fact, similar to a septic poison. The tissues which were especially liable to be affected were the fibrous tissues, such as the synovial membranes, fascia about the tendons, the pericardial and subendocardial layer of fibrous tissue. So also the serous membranes, the pericardium, pleura, and possibly very occasionally the peritoneum. So also an affection of the parenchyma of the lung, giving rise to a form of pneumonia had been described. The mucous membranes may be attacked, producing bronchitis, tonsillitis, or the skin, giving rise to erythema marginalis and nodosum, so also purpura rheumatica. The nervous system with symptoms of chorea, delirium (cerebral rheumatism), and this sometimes without pericarditis. All these phases of the disease are much modified by the periods of life; thus, in the rheumatism of early life all the morbid symptoms may be present, without any apparent arthritic trouble, and this, which must be considered the simplest expression of true rheumatism, should be taken as the typical form, and that found in later life as a modification of this. Our general ideas concerning rheumatism are reversed; we take the form found in the adult as the normal type, whereas we should take that of the child as our type. In the rheumatism found in childhood the arthritis is not the chief symptom as is the case in the adult, and, in fact, it may be entirely wanting. The endocardial affection is a much more important one, and in children this is at its maximum of intensity. As age advances, the endocardial affection becomes less important and the arthritis more important, and in later life the arthritis alone remains. So also the erythema and chorea diminish and die out as adult life comes on. In the same way pericardial and pleuritic mischief diminish as life advances, and, finally, the arthritis alone survives as the symptom of rheumatism. In children many of the signs of rheumatism may be present without arthritis, or the joint affection may be so slight as completely to escape observation. Possibly a slight stiffness or some transient discomfort about one or more joints, and this without any swelling or redness; it may be limited to one joint, possibly a slight stiff neck, or what is most common, a slight stiffness of the hamstring muscles. With this apparent absence of joint affection the most serious mischief may be going on in the cardiac structures. There may be endocardial mischief with chorea and subcutaneous nodules, and these may pass unnoticed, because no arthritic trouble happens to be present, and the cardiac mischief may give rise to little or no symptom at first. The points in the rheumatism of children are the chorea, the nodules, and the erythema, also peri-and endocardial mischief. These may occur separately, or may be all present together. At one time there may be endocardial mischief, and this may be followed by arthritic disease. These, which are the characteristics of the rheumatism of early childhood cease as life advances. Still another feature of early rheumatism is the greater subacuteness of the pyrexia; the temperature often not being over 101 or 102, and is rarely above this; being subacute, it is also very persistent, running on for weeks and weeks, at times disappearing, and then relapsing again. There is never hyperpyrexia, or, at least, we have never seen a case, that is, of hyperpyrexia which ended fatally, as sometimes occurs in adult life; hyperpyrexia is probably unknown in the rheumatic affections of children. Another very interesting feature in early rheumatism is the development of subcutaneous nodules. This is not a mere fancy, as some have supposed, but of great practical importance. When present, they are connected with the tendons or fasciæ, and are strongly indicative of cardiac trouble. There is the closest possible association between endo-and pericarditis and these nodules. Their presence is almost in itself diagnostic of heart disease, and this form of endocardial lesion is exceptionally fatal. In twenty-eight cases collected by Dr. Barlow of morbus cordis under these circumstances, eight proved fatal. This condition of the muscles and fasciæ is much the same as exists in the cardiac valves. There is the same proliferation or penetration of fibrous tissue with leucocytes. In the early stages this condition may probably disappear without leaving a trace behind in the muscles or fasciæ, and it is also probable that the same may occur with regard to the valves of the heart. The same condition of small nodules may be found in the pericardium itself. Acute rheumatism may begin at a very early age. The earliest case traced was about three months old. This child, who was under our care this year, was a small boy who, when three months old, had what the doctor called gout, that is, he was always crying, and the joints were swollen, tender, and stiff. When four and a-half years old he had an attack of convulsions, followed by paraplegia; he recovered very gradually, and when seen six months later he had a loud mitral murmur and extreme irregularity of the heart's action, in fact, he had probably suffered from embolic hemiplegia. Another child seen at the age of eleven months undoubtedly had rheumatism with stiffness of the joints. With regard to the treatment, there was an old adage which said that a patient required at least six weeks before recovering. When the alkali treatment first came into use excellent results ensued, at least, as far as the cardiac disease was concerned, but there was not much relief from pain or fever. Since the salicylates have come into general use, the results on the pain and fever have been very satisfactory; but the influence on the peri-and endocardium is very questionable, and statistics give no improvement in this direction. An investigation committee, a few months ago, published the following as the result of a very extensive inquiry: With salicylates alone the average duration of the disease was 19 days, with 8 days fever, and 10 days pain. Under the alkali treatment there was 36 days disease, with 13 days fever and 19 days pain. With the two combined, 34 days disease, with 10 days fever and 15 days pain. With salicylates and iodides combined, 46 days disease, with 17 days fever and 26 days pain.

